# Diving deeper into the underlying white shark behaviors at Guadalupe Island, Mexico

**DOI:** 10.1002/ece3.8178

**Published:** 2021-10-18

**Authors:** Marc Aquino‐Baleytó, Vianey Leos‐Barajas, Timo Adam, Mauricio Hoyos‐Padilla, Omar Santana‐Morales, Felipe Galván‐Magaña, Rogelio González‐Armas, Christopher G. Lowe, James T. Ketchum, Héctor Villalobos

**Affiliations:** ^1^ Instituto Politécnico Nacional, Centro Interdisciplinario de Ciencias Marinas La Paz Mexico; ^2^ University of Toronto Toronto ON Canada; ^3^ University of St Andrews St Andrews UK; ^4^ Pelagios‐Kakunjá La Paz Mexico; ^5^ Ecocimati AC Ensenada Mexico; ^6^ Department of Biological Sciences California State University Long Beach Long Beach California USA

**Keywords:** Bayesian inference, behavioral states, energy costs, movement strategies, telemetry

## Abstract

1. Fine‐scale movement patterns are driven by both biotic (hunting, physiological needs) and abiotic (environmental conditions) factors. The energy balance governs all movement‐related strategic decisions.
2. Marine environments can be better understood by considering the vertical component. From 24 acoustic trackings of 10 white sharks in Guadalupe Island, this study linked, for the first time, horizontal and vertical movement data and inferred six different behavioral states along with movement states, through the use of hidden Markov models, which allowed to draw a comprehensive picture of white shark behavior.
3. Traveling was the most frequent state of behavior for white sharks, carried out mainly at night and twilight. In contrast, area‐restricted searching was the least used, occurring primarily in daylight hours.
4. Time of day, distance to shore, total shark length, and, to a lesser extent, tide phase affected behavioral states. Chumming activity reversed, in the short term and in a nonpermanent way, the behavioral pattern to a general diel vertical pattern.

## Graphical abstract

This study linked horizontal and vertical movements of white sharks from active tracking data and obtained six different behavioral states through the use of hidden Markov models.

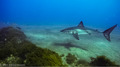

## 1 INTRODUCTION

How animals use a set of physical characteristics and resources in a habitat, together with the time over which they use them, is known as habitat use (Hall et al., 1997). Energy is the most precious asset in an ecosystem (Lawson et al., 2019), such that an animal must always be concerned with conserving it by balancing its acquisition and loss (MacArthur & Pianka, 1966) while minimizing predation risk (Bartumeus & Catalan, 2009). Consequently, animals must make different decisions throughout the day, optimizing their energy use (e.g., whether to hunt, take refuge, socialize for reproductive purposes or not, and rest) (Heithaus et al., 2001; Papastamatiou et al., 2011). This will lead to specific patterns of activity depending on the physical conditions (tides, time of day, temperature, wind, vegetation, currents, bathymetry, etc.) and external biological conditions (distribution and density of their predators and prey), as well as physiological requirements, at a given time (Houston & McNamara, 2014). In general terms, the more linear movements (and fewer directional turns) an animal employs, the more energy cost‐effective it can be (Wilson et al., 2013). However, in contrast to the terrestrial environment, the marine environment has a third dimension: depth. Thus, for shark behavior studies, it is essential to analyze types of movement in terms of both their horizontal and vertical components, which will allow us to characterize population trends, intra‐ and interspecific interactions (Langrock et al., 2012), and their distributions in space and time, ultimately improving conservation and management efforts (Bauer & Klaassen, 2013).

On the horizontal plane, we can distinguish between greater or lesser degrees of directed movements (patrol, displacement) and random movements. Some models point to different strategies for optimizing energy when searching for food based on straight movements interspersed with turns. Thus, we see Brownian motion for uncorrelated random movements at small scales and in the presence of concentrated prey; correlated random walks when adding directional persistence; Levy flight for more dispersed prey and larger spatial scales; and finally, Levy modulated correlated random walks under a combination of the previous conditions (Bartumeus & Catalan, 2005; Papastamatiou et al., 2011; Sims et al., 2012). However, other authors disagree with the classification above, alluding to the fact that these models do not take into account the higher energy cost involved in making a greater number of turns (Wilson et al., 2013) and that such a pattern would depend on a strategy of modular movements as an adaptation to spatial–temporal variation in environmental parameters, all within the context of the energy landscape (Shepard et al., 2013). On the vertical plane, we find more or less constant movements over time at specific depth ranges. For white sharks, such movements include patrolling in seal colonies (Goldman & Anderson, 1999) and another type referred to as diel vertical migration (DVM), which occurs when a predator follows the movement of the scattering layer, considered normal DVM when it is deeper during day, and reverse when it is deeper at night (Jorgensen et al., 2012; Weng et al., 2007).

In terms of hunting strategies, there are two general modes of behavior: “active searching/patrolling,” and “sit and wait” or area‐restricted searching (ARS). The former is when the predator moves around in its environment, either in a directed or random way, in search of prey. ARS is the equivalent of “sit and wait” for animals that cannot stop moving, consisting in the predator waiting for an extended period for the prey to enter the ambush area (Huey & Pianka, 1981; O’Brien et al., 1990; Towner et al., 2016). Different hunting modes can cause variation in trophic cascades at different levels in top‐down systems (Martin & Hammerschlag, 2012).

It is important to highlight the increasingly relevant effect of particular animal variability in studies of population dynamics due to individual specialization (promoted by inter‐ and intraspecific competition, availability of prey, and different physiological needs according to age, size, and sex). Such variability can occur in an animal's diet preferences, movement patterns, and other, more specific behaviors like habitat selection or hunting strategies (Bolnick et al., 2003; Matich et al., 2011; Towner et al., 2016). For example, while adult white sharks *Carcharodon carcharias* are mainly aggregated and seasonally resident near coastal or oceanic pinniped colonies in temperate waters, juveniles are more dispersed along the coast or around continental shelf islands (Domeier et al., 2012; Weng et al., 2007).

Guadalupe Island is considered a place of aggregation for one of the most important white shark populations in the eastern Pacific, which shows a high degree of fidelity to the site. For this reason, this location is also a target of cage‐diving tourism. The effect of chumming used as an attraction method can temporarily modify the natural behavior of the animals and hinder their study (Huveneers et al., 2013). White sharks can be observed throughout the year, with a seasonal peak between autumn and winter (Domeier & Nasby‐Lucas, 2008; Hoyos‐Padilla et al., 2016). In addition, on the island, there is an important community of pinnipeds, including the northern elephant seal (*Mirounga angustirostris*), the Guadalupe fur seal (*Arctocephalus philippii townsendi*), and the California sea lion (*Zalophus californianus*) that the white shark feeds on. These pinniped populations are distributed in different colonies around the island (Gallo‐Reynoso et al., 2005; Gallo‐Reynoso, Le Boeuf, et al., 2005).

Unlike many other white shark aggregation sites, Guadalupe Island is in a fully oceanic environment, which is why it plays a very important role in marine life in the same way as other oceanic islands and seamounts. Specifically, the island can provide predictable and productive feeding habitats, favoring primary and secondary production by island mass effects (Doty & Oguri, 1956); resting habitats and nurseries, offering refuge from predators and other disturbances; and navigation landmarks with unique acoustic, magnetic, chemical, thermal, visual, and hydrodynamic signals (Silva, 2016). One of the specific differences of oceanic islands compared with coastal islands in terms of foraging is that DVM of the deep scattering layer (DSL) takes place in closer proximity to the former; this introduces another set of potential prey, such as squids or deep‐sea fishes, that would otherwise require diving deeper (down to 400–500 m); such deep diving would cause stress because of the presence of cooler waters and the oxygen minimum zone (0.7–1.5 ml/L dissolved oxygen (DO)) located between 225 and 300 m in the eastern Pacific waters (Nasby‐Lucas et al., 2009). While the role of the island for this white shark population is unclear, evidence in previous studies showed that it can serve as a secondary nursery area for juveniles and as a feeding area for adults, which benefit from the pinniped colonies (mainly the northern elephant seal), although the frequency of attacks on them (seen at the surface) is not as high as in other areas such as California, which could be due to the excellent water clarity that allows ambush in deep waters (Domeier et al., 2012; Hoyos‐Padilla et al., 2016; Skomal et al., 2015). Moreover, according to new evidence (Becerril‐García et al., 2020; Le Croizier et al., 2020; Papastamatiou et al., 2020), Guadalupe Island could be more important than previously thought in terms of access to mesopelagic prey, acting as a barrier trap and hindering their dispersion.

To analyze the movement patterns of white sharks at Guadalupe Island, we use hidden Markov models (HMMs) and their extensions (Hooten et al., 2017; Zucchini et al., 2016). HMMs are an increasingly common and powerful statistical tool used in movement ecology to identify movement patterns that can serve as reliable proxies of behaviors of interest and to further identify key drivers of animal movements (Morales et al., 2004; Patterson et al., 2009, 2017). Such models have been used to model white shark movements in South Africa (Towner et al., 2016), juvenile white shark movements in eastern Australia (Bruce et al., 2019), and to identify activity patterns of a variety of other shark species (Adam et al., 2019; Papastamatiou, Iosilevskii, et al., 2018; Papastamatiou, Watanabe, et al., 2018). Their application extends to terrestrial systems, aerial systems, and general marine systems, for example, those including other fish and marine mammals.

One of the key qualities of HMMs is that they can be easily extended to capture movement patterns as a composition of multiple data streams (DeRuiter et al., 2017; McClintock et al., 2013; Schliehe‐Dieks et al., 2012). For instance, some devices can collect positional data, depth measurements, and acceleration data, among other data types, and we may want to use all available data streams to construct joint movement patterns of interest. However, one of the challenges in the application of HMMs, and most other common statistical models in movement ecology, is the key desire to know *how many* different behaviors an animal exhibited. This issue of the *number* of behaviors is further exacerbated by the inclusion of multiple data streams. Some of the key challenges are outlined in Pohle et al. (2017) and discussed in Li and Bolker (2017). In general, the collection of more data streams and fine‐scale movement data requires more “movement patterns” to capture the structure of the data, even if the number of behaviors of interest is small. However, we posit that the mismatch between captured movement patterns and behaviors of interest need not be an issue, as long as we can define behaviors of interest as (possible) compositions of the movement patterns (Adam et al., 2019; Leos‐Barajas et al., 2016, 2017; Pirotta et al., 2018).

We take this approach to analyze white shark movement data. For each data stream (vertical and horizontal, *d* and *p*, respectively), we identify $N_{d}\in N$ and $N_{p}\in N$ fine‐scale movement patterns for depth and positional data that are necessary to capture the structure of the data but specify *K_d_* < *N_d_* and *K_p_* < *N_p_* behaviors of interest that are (possible) compositions of the fine‐scale movement process. This allows us to infer the effects of environmental covariates, physiological features, and time of day on the behavioral processes of interest while still capturing the dependence structure and evolution of the fine‐scale movement process. We analyze each movement direction separately and combine the state results to gain an understanding of what the white sharks did as a collective group around Guadalupe Island. Model inference is conducted in a Bayesian framework using the software Stan (Carpenter et al., 2017).

We outline first the methodology used at the study site, how the sharks were monitored, and the HMM framework used to analyze the data set. Next, we present the main results of both analyses in the context of the model output and take a closer look at the white shark movement patterns around Guadalupe Island. We conclude with a discussion of the overall results, indicating the particularities expected for this oceanic region, the factors influencing movements, and the effect of ecotourism on white shark behaviors, finishing by the outlining of future work.

## 2 MATERIALS AND METHODS

### 2.1 Study site

**FIGURE 1:**
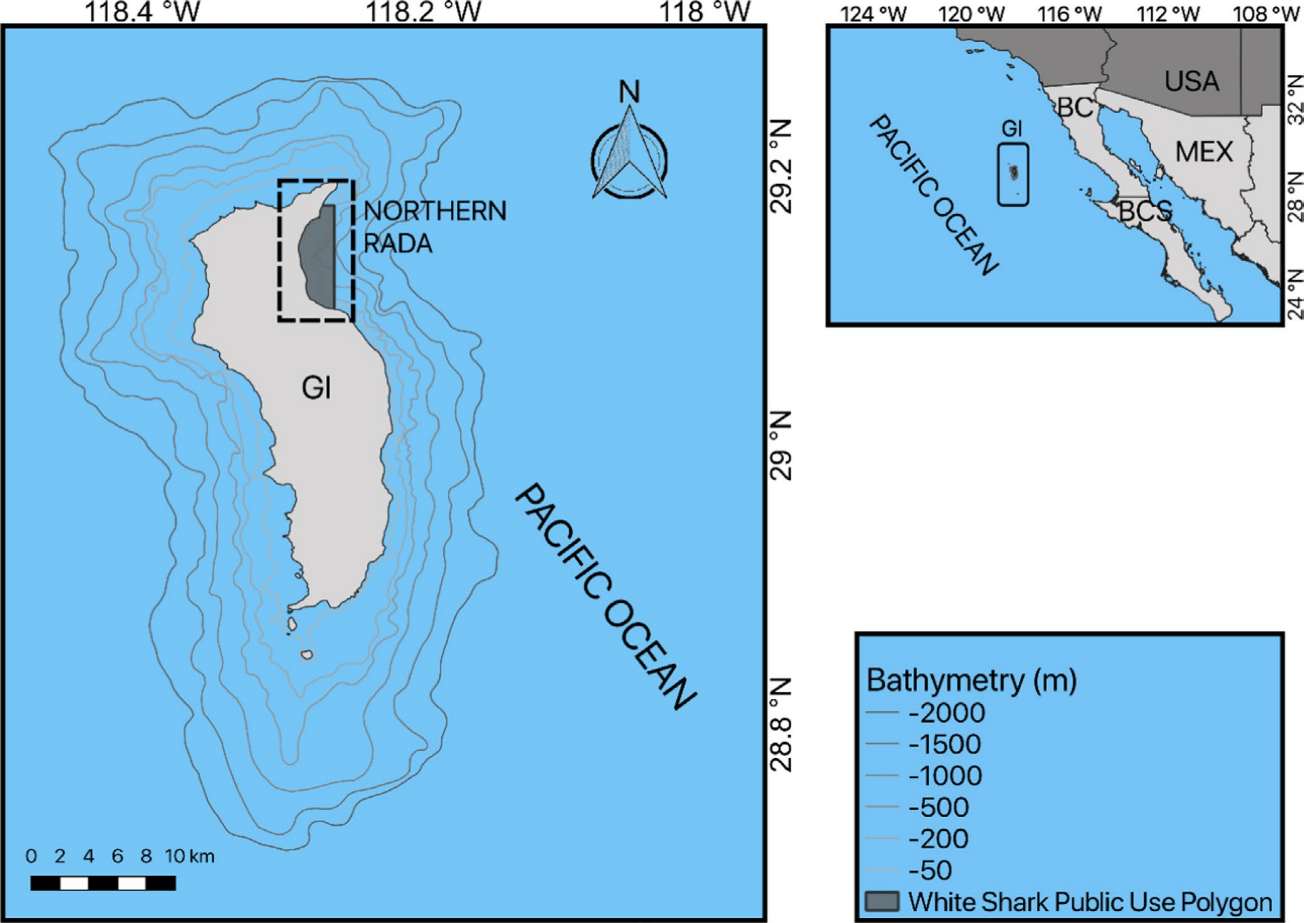
Macrolocalization (top right) of Guadalupe Island (GI) in the Mexican Pacific, west of Baja California peninsula. The detailed map of GI (left panel) includes the bathymetry down to the 2000 m isobath and highlights the Northern Rada and the White Shark Public Use Polygon (dark gray) where tourist boats operate. BC and BCS stand for Baja California and Baja California Sur, respectively

Guadalupe Island (29.0528°N, 118.3041°W) is located in the southern region of the California Current, 240 km off the western coast of the Baja California peninsula, Mexico (Figure 1). It is a volcanic cone rising 1300 m above sea level with a length of 36 km (oriented in the N–S direction) and width of 12 km (W–E) (García‐Gutiérrez et al., 2005). The eastern side is characterized by an abrupt change in bathymetry, with an average slope of 70° and deep underwater canyons extending to a 3600 m depth (Delgado‐Argote et al., 1993). In contrast, the southern and western sides have a platform approximately 4 km wide and 200 m deep. Under the cliffs, there are large blocks of rock detached from the surface up to 12 m high; these blocks are followed by sand plains with stepped slopes that extend to greater depths and are interspersed with rocky areas. The prevailing northwesterly winds affect the north and west of the island by dragging the swell from the Northeast Pacific, leaving the eastern area sheltered for the most part. In the Northern Rada, up to seven cage dive operators are active from 7 a.m. to 6 p.m. The boats anchor 500 m apart from each other, spreading along a 3.4‐km line over a depth range of 70–80 m inside the White Shark Public Use Polygon (Figure 1). Observers on board tourist boats are routinely in charge of monitoring shark behaviors, especially in relation to chumming (2021). Observers also register the number of boats operating every day.

### 2.2 Active tracking

Active tracking of white sharks took place mostly during September and October, 2015, through 2019. A total of 325 tracking hours on 10 different individuals were recorded. Underwater images of each white shark specimen were taken for identification purposes and sexing, while size was estimated by comparison with the work boat. Maturity status was determined as proposed by Bruce and Bradford (2012), that is, 1.75–3 m (juveniles), 3–3.5 m (subadult males), 3–4.5 m (subadult females), >3.5 m (adult males), and >4.5 m (adult females). Tagging procedures and ethics followed an animal care protocol (Protocol number 16022, UC Davis Institutional Animal Care and Use Committee) and authorized by the research permits provided by the General Directorate of Wildlife (SEMARNAT; permit numbers SGPA/DGVS/6948/15, 7052/16, 6673/17, 4284/18, and 6949/19).

To track sharks, Vemco V16TP‐6x tags with temperature (0–40°C) and pressure (depth 0–680 m) sensors were used. The tags were attached by nylon wire to an umbrella‐type plastic anchor and placed at the base of the dorsal fin via a pole spear, from the boat after attracting them with a piece of bait. The tag signals emitted every 3 s were received by a unidirectional VH110 hydrophone mounted on the side of the boat and connected to a VR100 receiver equipped with GPS, where the temperature, depth, position, and signal intensity data were recorded. We attempted to maintain a safe distance (between 100 and 500 m) from the animal to avoid influencing its behavior. Due to rough weather and concerns for crew safety, some trackings have to be interrupted and resumed later when conditions improved.

The acoustic propagation properties of the sound can vary according to different environmental conditions, such as wind, type of bottom, turbidity of the water, and depth. This could alter the real distances or depths of the sharks during data acquisition. A range test of the receiver was conducted to take this into account, mostly by keeping a proper distance to the tracked shark within reach of the receiver (<500 m) throughout all the tracking time.

### 2.3 Data preparation

Recorded tracks were exported to Excel, where data outside the sensors ranges as well as other incongruent information were eliminated. A finer filtering was done in the R environment (R Core Team, 2020), selecting records every 5 min, and searching for uninterrupted sequences of data with a minimum duration of 3 h. The distance to the shore of each data point in the sequences was calculated with custom R scripts. These sequences were used in the subsequent modeling. Tide level was obtained from monthly tidal calendars.

### 2.4 Multiscale hidden Markov models

We analyzed white shark movement patterns via an extended HMM framework in two contexts: (a) fitting an extended HMM to step lengths and turning angles (explained below), and (b) fitting an extended HMM to depths. In both analyses, we allowed for a mismatch between the number of movement states and the number of behaviors of interest in the HMM framework, in line with recent work in movement ecology (Adam et al., 2019; Leos‐Barajas et al., 2017; Pirotta et al., 2018), while also testing for potential environmental and physiological drivers of behavior.

**FIGURE 2:**
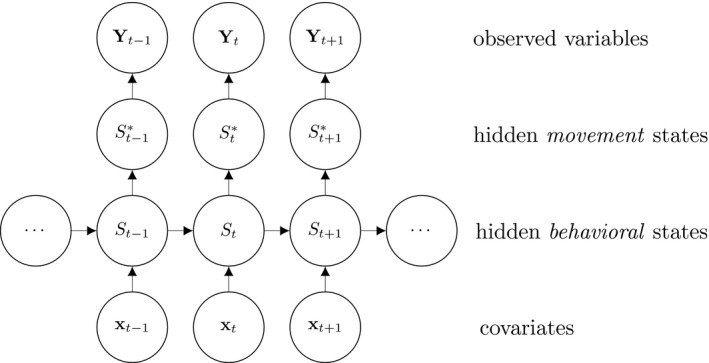
Dependence structure of the hierarchical HMM with a covariate‐dependent *behavioral* state process. At time *t*, the *behavioral* state process (*S_t_*) selects one of N* possible *movement* states $\left(S_{t}^{*}\right)$, which in turn determines the distribution for the observation process

A basic (discrete‐time, finite‐state) HMM is a doubly stochastic time series with an observed process (*Y_t_*) that depends on an underlying state process (*S_t_*), Figure 2. We assumed that *S_t_* can take a finite number *N *≥ 1 of states, such that we can also refer to this as an *N*‐state HMM. The observations ${\left\{Y_{t}\right\}}_{t=1}^{T}$ were taken to be conditionally independent given the states ${\left\{S_{t}\right\}}_{t=1}^{T}$ and were generated by so‐called state‐dependent distributions, *f*(*Y_t_* |*S_t_* = *n*), herein denoted by *f_n_* (·) for *n* ϵ {1,…*N*}. The evolution of states over time was governed by a Markov chain, that is, Pr(*S_t_* |*S_t_*_−1_, …, *S*_1_) = Pr(*S_t_* |*S_t_*_−1_), with transition probability matrix (t.p.m.) **Γ** = *γ_i_*_,_ *_j_*, where *γ_i_*_,_ *_j_* = Pr(*S_t_* = j|*S_t_*_−1_ = *i*) for *i*, *j* = 1, …, *N*, denotes the probability of switching from state *i* at time *t*−1 to state *j* at time *t*. Lastly, it was necessary to define the initial state distribution **δ** for the state process at time *t* = 1 with entries *δ_n_* = Pr(*S*_1_ = *i*), for *i* = 1, …, *N*, which denote the probabilities of the state process starting in state *n*.

In the specific application of animal movement, the different states serve as proxies for behaviors of interest, the t.p.m. tells us how the animals switch between distinct movement patterns, and the initial state distribution relays what the animals may have been doing when first observed. Covariates are commonly inserted into the state process equations (the t.p.m.), where they can be used to investigate how the probability of switching to a certain behavior is determined by potential environmental and physiological drivers. In the next section, we demonstrate how covariates may be included when the number of movement states differs from the number of behavioral states.

### 2.5 From movement to behavior

When applying HMMs in an unsupervised manner to animal movement data, there are two features of the data that we aim to capture: (a) the marginal distribution and (b) temporal dependence. In general, if we capture the marginal distribution well enough, we should obtain a density curve that captures the histogram of the movement data well across all data streams. However, in many cases, the number of states that are needed to capture the data patterns well can be much greater than would be biologically relevant (Langrock et al., 2018, 2018; Pohle et al., 2017). To address this issue, we extend the basic HMM so that there are two underlying state processes, a *movement* state process and a *behavioral* state process. We allow for the model to capture as many movements as necessary to model the data but combine movements to construct the behavior of interest. In this manner, one movement state can correspond to one behavioral state, or we can extend the framework so that multiple movement states correspond to a single, larger behavioral state of interest. This extension plays an important role when attempting to identify potential environmental and physiological drivers of behavior. Formally, we construct an HMM with a multiscale state process, hereafter denoted as hierarchical HMM, for simplicity.

#### 2.5.1 Horizontal movements

For the horizontal movement analysis, we first transform positional data into step lengths and turning angles using Gamma and Von Mises distribution, respectively (Hooten et al., 2017). Step lengths were computed as Euclidean distances traveled within 5 min between two consecutive positions, while turning angles were computed as angles between two consecutive line segments. Most analyses of animal positional data attempt to broadly identify ARS, traveling, and resting behaviors. For white sharks that never rest, in line with previous analyses (see Towner et al., 2016), we focus solely on ARS and traveling behaviors. These two categories roughly represent periods of large turning angles/small step lengths and directed travel/large step lengths, respectively. Generally, this would indicate that *N*_movement_ = 2 and *N*_behavioral_ = 2 thus implying a one‐to‐one relationship between movement and behavior. However, this is not the case for *some* of the time series, in particular the sharks that were tracked for a longer period of time (>16 h). Given the reality that some time series may be much shorter than others and thus contain much less information, we demonstrate how to allow for some sharks to follow a three‐state HMM (with two states indicative of varying traveling behaviors) and the other sharks to follow a two‐state HMM (with only one state relaying traveling behavior).

Let **Γ** *^B^* be a 2 × 2‐dimensional t.p.m. that models switching between ARS and general traveling behaviors. For the sharks where *N*_movement_ = *N*_behavioral_ = 2, the model setup is exactly like a two‐state HMM (Zucchini et al., 2016). For the sharks with three movement states, that is, *N*_movement_ = 3, we further define **Γ** *^M^* and **δ** *^M^*, the fine‐scale movement t.p.m. and initial state distribution for the two traveling movement patterns. To allow for three fine‐scale movement states but enforce only two *behavioral* states, we construct the t.p.m. in the following manner:

(1) $$\Gamma =\left(\begin{array}{ccc} \gamma_{1,1}^{B} & \gamma_{1,2}^{B}\delta_{1}^{M} & \gamma_{1,2}^{B}\delta_{2}^{M}\\ \gamma_{2,1}^{B} & \gamma_{2,2}^{B}\gamma_{1,1}^{M} & \gamma_{2,2}^{B}\gamma_{2,1}^{M}\\ \gamma_{2,1}^{B} & \gamma_{2,2}^{B}\gamma_{1,2}^{M} & \gamma_{2,2}^{B}\gamma_{2,2}^{M} \end{array}\right)\;\text{or more concisely as}\;\Gamma =\left(\begin{array}{ccc} \gamma_{1,1}^{B} & & \gamma_{1,2}^{B}\delta^{M}\\ \gamma_{2,1}^{B} & & \gamma_{2,2}^{B}\Gamma^{M} \end{array}\right)$$

In Equation (1), $\gamma_{2,1}^{B}$, for instance, refers to the probability of switching from behavioral state 2 to behavioral state 1, while $\gamma_{2,2}^{B}\gamma_{2,1}^{M}$ refers to the probability of switching from fine‐scale movement state 2 within behavioral state 2 to fine‐scale movement state 1 within behavioral state 2. $\gamma_{1,2}^{B}\delta_{1}^{M}$, to give another example, refers to the probability of switching from behavioral state 1 to behavioral state 2. This setup allows for a clear mechanism that governs the behavioral process, the entries of **Γ** *^B^*, but still models the switches between the two traveling movement patterns via the entries of **Γ** *^M^*. When testing for drivers of behavior, we allow for the entries of **Γ** *^B^* to be a function of covariates of interest but keep **Γ** *^M^* fixed.

#### 2.5.2 Vertical movements

The motivation for the analysis of white shark vertical movements is to identify differences in movements in three depth layers of the water column: (a) at the surface (~0–40 m), (b) at midrange depths (~40–80 m), and (c) at greater depths (~>80 m). The depths encompassed by the “surface” state are those at which the sharks are directly exposed to touristic activity when they occur in proximity to the boats. We partition the *N*_movement_ = 6 movement states into *N*_behavioral_ = 3 large‐scale behavioral processes, whose evolution over time is governed by *N*_behavioral_ × *N*_behavioral_ dimensional t.p.m., **Γ** *^B^*. Here, a Gamma distribution was used.

We combine two states to reflect the *shallow* depth movements, another two to reflect the *midrange* depth movements, and the last two to reflect *deep*‐water movements. Let **Γ** *^S^* reflect the movements across the shallow states, and the same for the other two states, **Γ** *^MR^* and **Γ** *^D^*, midrange and deep‐water movements, respectively. Let the initial state distributions **δ** *^S^* ^→^ *^MR^* reflect the initial distribution of the midrange depth process when transitioning from the shallow depth process and so on for the other possible transitions. Then, the overall t.p.m. for the depth process, **Γ**, is given as

$$\Gamma =\left(\begin{array}{ccc} \gamma_{1,1}^{B}\Gamma^{S} & \gamma_{1,2}^{B}\delta^{S\to MR} & \gamma_{1,3}^{B}\delta^{S\to D}\\ \gamma_{2,1}^{B}\delta^{MR\to S} & \gamma_{2,2}^{B}\Gamma^{\mathrm{MR}} & \gamma_{2,3}^{B}\delta^{MR\to D}\\ \gamma_{3,1}^{B}\delta^{D\to S} & \gamma_{3,2}^{B}\delta^{D\to MR} & \gamma_{3,3}^{B}\Gamma^{D} \end{array}\right).$$

#### 2.5.3 Covariates

As performed in Towner et al. (2016) and 2018 Papastamatiou, Watanabe, et al. (2018) we test for effects of tide level, shark length, and time of day, as well as distance to the shore, sex, and number of tourist boats during the tracking period, on the horizontal as well as vertical (in this study) state‐switching dynamics of the sharks. Let the vector of covariates be **x** *_c_* = {tide, time, length*, distance*, sex, boats number*}, where any covariate with an asterix has been transformed to be approximately centered at zero. In this case, $\beta_{0}^{\left(i,j\right)}$ reflects the baseline effect of the *ebb* tide for an 4‐m female shark approximately 400 m from the coast when there are 4 boats in the area. The tide level is denoted as 0 s or 1 s, and time is given by two trigonometric functions, namely, $\cos\left(2\pi t/1440\right)$ and $\sin\left(2\pi t/1440\right)$, which assume a daily periodicity, where $t\in \left\{0,\ldots ,1349\right\}$.

We then allow the entries $\gamma_{i,j}^{B}$, for $i,j\in \left\{1,\ldots ,N\right\}$, to be functions of environmental covariates of interest via a multinomial logistic link as follows:

$$\gamma_{i,j}^{B}\left(t\right)=\frac{\exp\left(\eta_{i,j}^{\left(t\right)}\right)}{\sum_{k=1}^{N}\exp\left(\eta_{i,k}^{\left(t\right)}\right)},\;\text{where}\;\eta_{i,j}^{\left(t\right)}=\left\{\begin{array}{cc} \beta_{0}^{\left(i,j\right)}+\sum_{c=1}^{C}\beta_{c}^{\left(i,j\right)}x_{c,t} & \text{if}i\neq j;\\ 0 & \text{otherwise}. \end{array}\right.$$

Although there were not many observations of high and low tide in the data set, these factors can also be included as covariates in similar analyses.

### 2.6 Inference

We conduct inference in a probabilistic framework to obtain distributions of all parameters of interest. The likelihood of the hierarchical HMM can be constructed in the same manner as that of the basic HMM. Let **P**(*y_t_*) be an *N*_movement_ × *N*_movement_ diagonal matrix with entries $P_{\mathrm{nn}}\left(y_{t}\right)=f_{n}\left(y_{t}\right)$ for $n\in \left\{1,\ldots ,N_{\text{movement}}\right\}$. The expanded t.p.m. given in the *Horizontal Movements* section that combines movement and behavior describes the evolution of the process of interest over time, and we further define **δ**_movement_ as the distribution of the state process at time *t* = 1, with entries $\delta_{n}=\mathrm{Pr}\left(S_{1}=n\right)$, for $n\in \left\{1,\ldots ,N_{\text{movement}}\right\}$. Given these three components, we can express the joint distribution of the observation process for a single time series as a matrix product,

$$f\left(y_{1},y_{2},y_{3},\ldots ,y_{T}\right)=\delta^{⊤}P\left(y_{1}\right)\prod_{t=2}^{T}\Gamma P\left(y_{t}\right)1^{⊤},$$

where $1^{⊤}$denotes a column vector of ones. If we assume independence across *W* individual tracks from M∈N sharks, we can write the joint distribution as a product, $f\left(y\right)=\prod_{w=1}^{W}f\left(y_{w}\right)$. We additionally take into account the individual‐level variation in the *M* shark's horizontal movements by allowing for the means of the state‐dependent step length distributions to differ among sharks, as this allows each shark to have slightly different estimates of “short” and “long” step lengths, a feature that is evident in the observed data.

To probabilistically express our uncertainty in the parameters of interest given the data, we use a Bayesian framework. We assign nonexchangeable prior distributions for the location parameters of the state‐dependent distributions and further impose ordering (Betancourt, 2017). The priors for the construction of the combined t.p.m. assume baseline persistence in behaviors over time, but $\beta_{c}^{\left(i,j\right)}$ are assigned normal distributions centered at zero so as to not *a priori* assume any type of effect of the environmental covariates. Given the full set of prior distributions and joint distribution of the observations, we can write the posterior distribution as follows:

$$p\left(\theta |y\right)\propto \left[\prod_{w=1}^{W}f\left(y_{w}|\theta \right)\right]\pi \left(\theta \right),$$

where **θ** represents a vector that includes all parameters requiring estimation. As *p*(**θ**|**y**) is not available in closed form, we use the software Stan to draw samples from the joint posterior distribution (Carpenter et al., 2017).

### 2.7 State decoding

In this application, where the behavioral modes were not observed and, as a consequence, no labeled data are available, estimation of the underlying state sequence is not the focus of the analysis but rather a convenient by‐product of the HMM framework, as we do not measure the efficacy of the model by the ability of the states to capture specific behaviors of interest. To obtain draws from the joint posterior distribution of the state process, we use the forward‐filtering backward‐sampling (FFBS) algorithm (Frühwirth‐Schnatter et al., 2018).

## 3 RESULTS

We analyzed a total of 24 tracking segments from 10 individual sharks consisting of four adult males, one adult female, four subadult females, and one juvenile female between 2015 and 2019 (Table 1), each with at least ~3 h of tracking data and a position collected every 5 min. The tracking segments used for the analysis ranged from 3 to 23.9 h in length for a corresponding 35–287 observations across each time series.

**TABLE 1:** Tracking specifications for 10 white sharks tagged at Guadalupe Island from 7 September 2015 to 8 October 2019. Tracking segments refers to individual noncontinuous monitoring time period

Shark ID	Sex	TL (cm)	Start date	End date	Tracking segments	Tracking hours
WS1	M	400	7/9/15	26/9/15	4	49.5
WS2	F	400	13/9/15	14/10/15	2	36
WS3	M	450	21/9/15	22/9/15	1	24
WS4	M	400	3/10/15	6/10/15	2	40.5
WS5	F	400	24/8/16	27/9/16	5	61.7
WS6	M	500	28/9/16	30/9/16	1	39.5
WS7	F	400	15/10/17	17/10/17	4	19
WS8	F	300	14/10/18	14/10/18	1	11
WS9	F	500	26/9/19	28/9/19	2	21
WS10	F	400	4/10/19	8/10/19	2	23
Total						325.2

### 3.1 Horizontal movements

**FIGURE 3:**
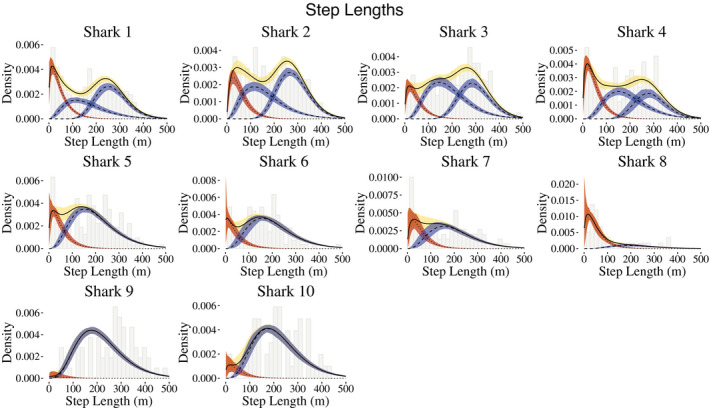
Individual‐level fitted state‐dependent distributions of step lengths associated with behavioral states 1 (red) and 2 (blue), along with the marginal distribution, weighted by the proportion of observations connected to each state. Mean posterior draws are indicated by lines, and 95% credible intervals are depicted by shading

**FIGURE 4:**
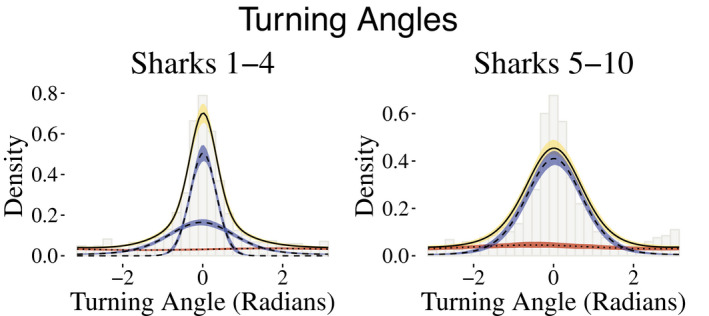
Group‐level fitted state‐dependent distributions of turning angles associated with behavioral states 1 (red) and 2 (blue), along with the marginal distribution, weighted by the proportion of observations connected to each state. Mean posterior draws are indicated by lines, and 95% credible intervals are depicted by shading

We used *N*_behavioral_ = 2 states for the behavioral process to capture periods in which the sharks were conducting ARS behavior or traveling behavior, with the former exemplified by short distances and large turning angles and the latter corresponding to longer step lengths and directed traveling (angles of approximately zero). Because many more data were available for some sharks than for others, a shark's horizontal movement track was modeled via 2 or 3 movement states, and the state‐dependent distributions for step lengths were allowed to vary by individual. For the shark tracks with 3 movement states, there are 2 movement states that fall under the umbrella of traveling behavior, that is, longer steps and directed travel. The fitted state‐dependent distributions of step lengths and turning angles are illustrated in Figures 3 and 4, respectively.

Across all sharks, the mean point estimates of step lengths for behavioral state 1 ranged from 46 to 55 m. For the four sharks with two states encompassing general traveling behavior, the point estimates for the means of the step length distributions ranged from 162 to 190 m and 267 to 298 m, respectively. Sharks with a single traveling behavior had means for the step length distributions ranging from 198 to 219 m. The turning angle distributions generally reflect directional persistence (behavioral state 2) and uniform turning angles (behavioral state 1).

The state‐switching process varied according to the length of the shark, distance to shore and time of day. For time of day, certain hours coincided mostly with a particular tide, either ebb or flood. We chosen 6:00, 12:00, 18:00, and 0:00 h as equidistant periods of time near the start and end times of tourist activity (7:00–19:00 h) to achieve greater representativeness. To visualize the impacts that these covariates have on the state‐switching dynamics, we computed the (pseudo‐) stationary distributions, that is, the marginal probability of the state as a function of the covariate values.

**FIGURE 5:**
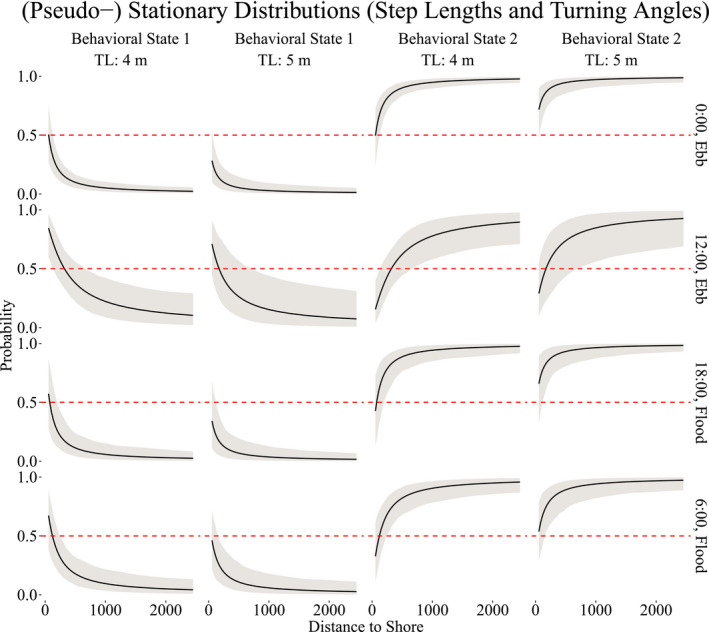
(Pseudo‐) stationary distributions of the behavioral processes as functions of distance to shore, time of day, ebb and flood tides, and size of the shark (in m), for behavioral states 1 (ARS) and 2 (traveling). The dashed line indicates a 50% probability

Figure 5 demonstrates that sharks are more likely to be in behavioral state 1 (ARS) when closer to shore, particularly during mid‐day. At midnight, the sharks are <50% likely to be engaged in ARS, even when closer to shore, and much more likely to be conducting general traveling behavior (behavioral state 2) at any distance. Slight differences are evident across the two sizes of sharks, 4 and 5 m, as shown in Figure 5, with smaller sharks more likely than larger sharks to be engaged in ARS behavior at any time of day when closer to shore.

### 3.2 Vertical movements

**FIGURE 6:**
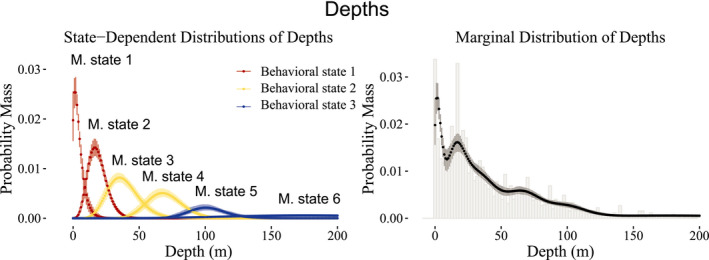
(Left panel) Point‐wise 95% credible intervals and posterior means of the population‐level fitted movement state‐dependent distributions of depths associated with behavioral states 1 (red), 2 (yellow), and 3 (blue), weighted by the proportion of observations associated with each movement (and subsequently behavioral) state. (Right panel) Histogram of depths (rounded to the nearest whole number) along with point‐wise 95% credible intervals of the marginal distribution

**FIGURE 7:**
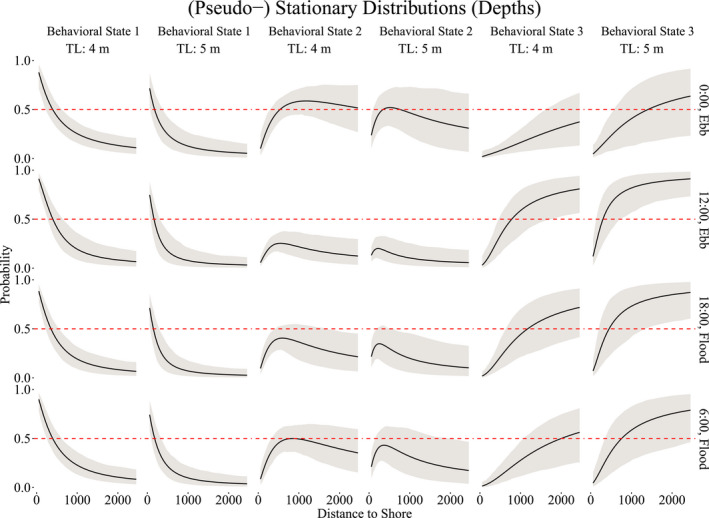
(Pseudo‐) stationary distributions of the behavior processes as functions of distance to shore, time of day, ebb and flood tides, and size of the shark (in m), for behavioral states 1 (surface depth), 2 (midrange depth), and 3 (deep depth). The dashed line indicates a 50% probability

To model vertical movements, we pooled the data across all sharks to construct the production and behavioral states needed to capture the marginal distribution and auto‐correlation structure, with the intention of creating behavioral states that generally reflected *surface*, *midrange*, and *deep* depths. Figure 6 illustrates the six movement states required to capture the marginal distribution of depth. The behavioral state‐switching dynamics were governed by distance to shore, length of the shark, and time of day, with a slight effect of tide on the behavioral state related to shallow depths only, as demonstrated in Figure 7. When the sharks are closer to shore, at any time of day, they are likely to be in shallower waters, as expected due to the bathymetry of Guadalupe Island. The probability that larger sharks (~5 m) are in shallower waters fell below 50% when more than 500 m from the shore, while for smaller sharks (~4 m), this occurred when more than 1000 m from the shore. Overall, the smaller sharks had a higher probability of being in shallower waters than the larger sharks across all times of day when within of shore. Midrange depths were more likely to be inhabited in the evening hours than at mid‐day across all sizes of sharks, while deeper waters were more likely to be inhabited at mid‐day than in the evening. In particular, during the mid‐day hours, sharks were more likely to be either at shallow or deep depths, rather than midrange depths.

### 3.3 Joint state results

**FIGURE 8:**
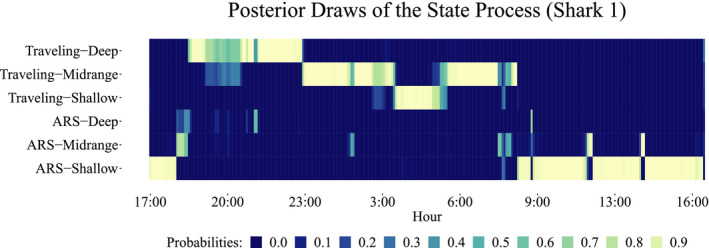
100 joint posterior draws of the underlying state process at each point in time for track 1 of shark 1. Colors reflect the proportion of draws connected to each of the six behavioral state combinations

For both vertical and horizontal displacements, the analyses demonstrated that the movement patterns varied according to time of day, length of the shark, and distance to shore. A slight effect of tide, differentiating between ebb and flood periods at shallow depths, was also observed in the vertical movement analysis. To visualize the white shark pattern results jointly, we implement the FFBS procedure to sample from the joint posterior distributions of the underlying behavioral state processes from both the vertical and horizontal movement analyses, *p*(**S**|**y**), for each track (Frühwirth‐Schnatter et al., 2018). For the horizontal movement analysis, the behavioral state process can take on values of {1,2} (proxies for ARS and general traveling behaviors), while in the vertical movement analysis, the state process takes values of {1,2,3} (proxies for shallow, midrange, and deep depths). Combining the posterior draws of the two behavioral state processes, we generate combined state results [{1,1},{1,2},{1,3},{2,1},{2,2},{2,3}], such that {*i*,*j*} denotes the *i*‐th behavioral state for horizontal movements and the *j*‐th behavioral state for the vertical movement process. Generally, these states reflect (in the order presented) {“ARS–Shallow,” “ARS–Midrange,” “ARS–Deep,” “Traveling–Shallow,” “Traveling–Midrange,” and “Traveling–Deep”}. To demonstrate the inherent variability associated with the state decoding process (i.e., assigning a state to an observation), Figure 8 shows 100 joint posterior draws from the state process using the FFBS algorithm for one track from shark 1.

**FIGURE 9:**
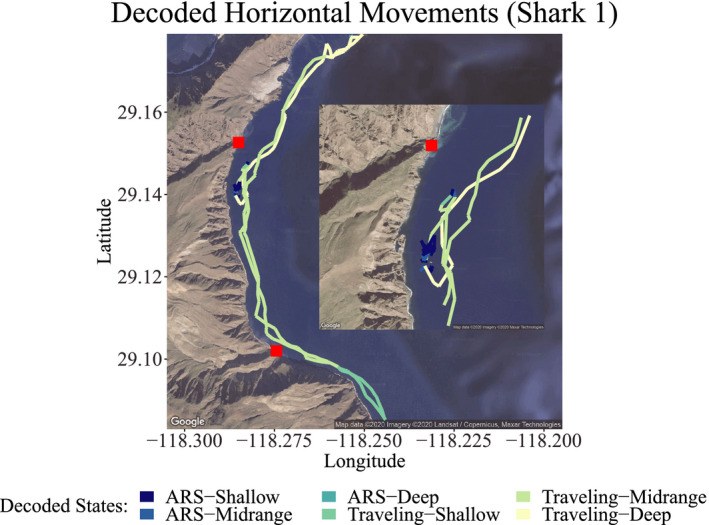
State decoding of one track from shark 1 around Guadalupe Island. Only the state with the highest probability at each time *t* is visualized here, with full state uncertainty illustrated in Figure 8. Red squares denote the main northern elephant seal colonies in the area

For shark 1 (Figure 9), during track 1, ARS‐Shallow and Traveling‐Shallow were the most geographically restricted behaviors, the first occurring in the central area of Northern Rada (a core tourist activity area) and the second, south of the bay. However, Traveling‐Deep and Traveling‐Midrange were the most commonly observed behaviors, with the former being observed mainly between the core area of tourist activity and the northernmost area and the second having the most widespread distribution.

**FIGURE 10:**
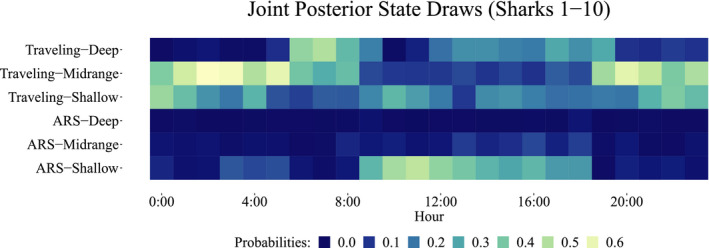
All posterior draws of the state process aggregated by hour for all sharks. Yellow indicates the most likely states in a given hour, while blue indicates that the event did not occur often

When did the sharks show each of the behavioral strategies? In Figure 10, we can see all shark joint posterior state draws by hour of occurrence. Then, ARS–Shallow occurred mainly between 9:00 and 18:00 h with high probability range (0.3–0.6), coinciding with the hours of tourist activity. ARS–Midrange took place mainly between 8:00 and 18:00 h with lower probability range (0–0.2). ARS–Deep was very little performed across all time, between 0–0.1 range. Traveling–Shallow occurred widespread in all daytime with a 0.3–0.5 probability range. Traveling–Midrange occurred from 19:00 to 8:00 with 0.4–0.6 probability range. Finally, Traveling–Deep state prevailed between 6:00 to 19:00 h and 0.2–0.6 probability range. Therefore, in general terms, we can see how ARS states took place primarily in daylight hours, except for ARS–Deep, which was the less performed at any time. Traveling states primarily occurred at night and twilight, except for Traveling–Deep, which was more likely to occurring during the daytime.

**FIGURE 11:**
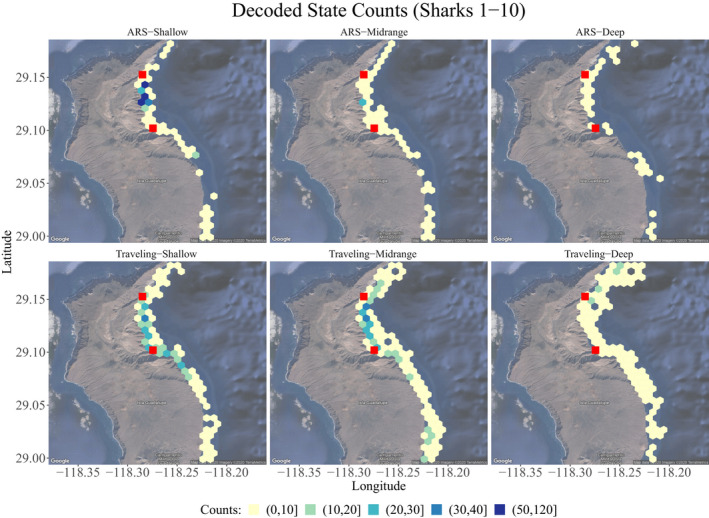
White shark state counts across the region. Colors indicate an increasing range from white to dark blue. Red squares denote the main northern elephant seal colonies in the area

Where and how did the sharks move? Figure 11 shows the spatial distribution of the different behavioral states as a function of their intensity of occurrence expressed in counts. ARS‐Shallow obtained the highest count of all the states, (20, 30]–(50, 120], concentrated in the area of ecotourism. In addition, this behavioral state was also observed, but to a lesser extent, along the coast, within little distance from it. ARS–Midrange presented a low count (0, 10] and occurred homogeneously near the coast, except in the central area of Northern Rada, where it was farthest from the coast, and in the second third of the east. ARS‐Deep also had a low count (0, 10] and occurred more irregularly along and near the coast. Traveling–Shallow was more common (10, 20]–(30, 40) in the central area of Northern Rada and south of it, close to the coast, and showed a lower count (0, 10] and more significant variability and coverage near the coast. Traveling‐Midrange was most common (10, 20]–(30, 40) in the central area of Northern Rada and closer to the coast. However, the distribution of its lowest counts (0, 10] had more considerable variability near the coast and covered more space. Traveling–Deep presented low counts (0, 10] and extended to a greater distance from the coast than the other states and homogeneously along the coast. Thus, the overview tells us that ARS states occurred more intensely in highly restricted areas, as expected. In contrast, the traveling states were characterized by occurring with greater intensity in larger areas, which is intuitive because they are movements with longer, linear steps.

How long did the sharks remain in each state, and what was the day/night contrast? As shown in Table 2, for the overall time, the most commonly used state was the Traveling–Midrange (36%), followed by Traveling–Shallow (25.5%), ARS–Shallow (17.2%), Traveling–Deep (16.3%), ARS–Midrange (4.3%), and, finally, ARS–Deep (0.5%). However, this order prevailed mostly at night; during the day, it changed drastically, with ARS–Shallow being the most common state (29.1%), followed by Traveling–Deep (25.3%), Traveling–Shallow (20.5%), and Traveling–Midrange (17.5%). The order of the last two states was consistent between the whole day and the night. Taking these results together and zooming out, we found that for both overall time, day, and night, the traveling state was always the most used, with frequencies of 77.8%, 61.4%, and 89.4%, respectively. ARS was the least used, with a frequency of 22.2% for overall time, 38.6% for daytime, and finally, 10.6% for night‐time.

**TABLE 2:** Point estimates of the proportion of time spent in each state across all sharks for three categories: Overall (all day), Day (6:00–18:00 h), and Night (19:00–6:00 h)

Proportion of time in each state						
ARS‐Shallow	ARS‐Midrange	ARS‐Deep	Traveling‐Shallow	Traveling‐Midrange	Traveling‐Deep	
Overall	0.1725	0.0437	0.0055	0.255	0.36	0.1633
Day	0.2911	0.0668	0.00767	0.2058	0.1751	0.2535
Night	0.0642	0.02249	0.0036	0.3	0.5289	0.081

## 4 DISCUSSION

Active tracking allows us to obtain data on fine‐scale animal movement. When performing such research in three‐dimensional spaces, such as the marine environment, we must also include the vertical component for a more accurate and holistic understanding. Movement patterns can vary depending on various factors, such as sex, size, age, life history, and energy requirements (Papastamatiou et al., 2011). On the other hand, the relationship between different movements and habitat will depend on various factors, such as the success of predation and the physiological limitations imposed by the physical environment in which the movement occurs (Patterson et al., 2009). The use of an HMM allows us to quantify and categorize these different behavioral strategies from animal time‐series data composed of continuous positional data collected at a fine temporal scale.

This study is the first of its kind to integrate the vertical depth component with commonly used horizontal movement patterns in white shark behavioral studies. Our results revealed two general types of behavior that corresponded to two main strategies: ARS, which may be more costly in terms of energy expended involving greater tortuosity, and traveling, which is more linear and therefore less costly. Traveling states can be considered a patrolling type of behavior when the conditions under which they occur better fit a hunting strategy. When the vertical component was incorporated, six different states/behaviors were obtained: ARS–Shallow, ARS–Midrange, ARS–Deep, Traveling–Shallow, Traveling–Midrange, and Traveling–Deep. These states and their transitions are affected by time of day, chumming, total shark length, and distance to shore. Slight differences were also observed between flood and ebb tides.

The time of day effect is explained by two approximations. Under natural conditions, the diel photoperiod is the most predominant driver of activity patterns in marine ecosystems. A Diel Vertical Pattern (DVP) that is shallower at night and deeper during the day is observed and considered normal DVM. Less often, the opposite, reverse vertical migration, is observed. On the one hand, this is considered to occur because of predators following DSL movements and avoiding other animals that prey on them (although this is not the case for white sharks, which are the top predator at Guadalupe Island) (Kronfeld‐Schor & Dayan, 2003). On the other hand, there may be physiological reasons due to different environmental conditions, such as temperature, salinity, light, density, and dissolved oxygen, in the water column (Afonso et al., 2014). In this sense, Traveling–Midrange and Traveling–Deep were the states that showed the greatest contrasts between day and night, although oppositely reflecting the pattern of DVM.

Tourism activity alters this natural diel pattern through the effect of chumming, which attracts sharks to surface waters in daylight hours (Huveneers et al., 2013, 2018). However, this change is also driven by environmental conditions (Pyle et al., 1996) in addition to the individual variability of sharks according to their preferences and physiological needs (Matich et al., 2011; Towner et al., 2016). Based on our direct observations, at an individual level, sharks approach tourist boats when they wish, completely ignoring them on other occasions. Especially if we take into account that ARS behavior carried out under tourist influence will be energetically more expensive than traveling behaviors, it is reasonable to expect sharks will return to their natural activities when they become aware of the unfavorable cost‐benefit balance (Wilson et al., 2013).

Shark movement varied according to shark size, as in Hoyos‐Padilla et al. (2016), with larger sharks swimming in open and deeper waters during the day and shallower during the night. Meanwhile, juveniles occupied shallower and coastal waters than adults throughout the observed time periods. This may be due to the adults thermoregulatory capacity, which allows them to better tolerate colder waters than juveniles (Hoyos‐Padilla et al., 2016), the heat loss due to higher juvenile body surface/volume relation (Block & Finnerty, 1994), and to different needs depending on maturity, with juveniles being unable to prey on large pinnipeds such as the northern elephant seal and, therefore, seeking their prey in more coastal and shallower waters while minimizing the risk of being preyed upon by adults by choosing different environments (Klimley, 1985). However, it must be pointed out that male sharks with a length of 3.5 m are already considered adults, while female sharks are considered adults beginning at a length of 4.5 m (Bruce & Bradford, 2012), so in our case, we could not detect differences between sexual maturity states.

Tidal effects are more substantial in coastal and shallower environments, where tides can influence the habitat use and behavior of certain species (Nagelkerken et al., 2008). In the Farallon islands, where the Northern elephant seal attacks occur close to the surface, Anderson et al. (1996) found that at high tides, the probability of more pinniped prey in the water increased, leading to a greater maximum number of predation events. The fact that tides had only a minor effect in our study is consistent with expectations for more oceanic environments, where tides could affect the cyclical pattern of the DSL (Afonso et al., 2014) by interacting with the structure of the water column. Shepard et al. (2006) found a tidal pattern of vertical movement in basking shark (*Cetorhinus maximus*), which as a filterer have a stronger relation with DSL than white sharks. In addition, it would be interesting to examine current patterns and thermocline depth at different tidal phases, since sharks could benefit from them, for example, by saving energy when moving with the current or by obtaining better oxygenation and olfactory traces when moving against it. Unfortunately, the data obtained from the tests carried out in this study did not reflect any correlation in this regard, and a more comprehensive effort is needed.

ARS was presumably most costly in terms of energy and, therefore the least used state of movement (22% of the overall time), with exception of the ARS‐Shallow state, during daytime hours possibly reflects the attraction effect of tourism activity. It accounted for 29.1% of daytime total proportion, as opposed to 6.4% of the night hours. ARS–Midrange was mainly observed between 08:00 and 18:00 h (indirect influence of the tourist activity, where sharks made detours in the vicinity of the boats anchored at a 70–80 m depth without directly interacting with bait or cages). ARS–Deep was concentrated mainly in the deep waters of ecotourism area and was generally less common. Possible reasons for this type of behavior could be the search for mesopelagic prey at depth. Given that this type of movement is more costly due to its higher degree of tortuosity (Wilson et al., 2013), and that to access them, they would have to expose themselves to the influence of the minimum oxygen layer, it makes sense that this would be the least used behavior.

Traveling behavior, a single state that is potentially a composite of both traveling and patrolling, was the most common movements by sharks (77.8% of the overall time). These movements were the most widespread on the N‐S axis along the coast, as well as the most used in the areas farthest from the shore, indicating that offshore habitat use was proportional to depth range; Colefax et al. (2020) found similar behaviors in drone tracked white sharks in Australia. There are two reasons for choosing this type of strategy, according to the energy landscape concept, that is, the movement strategy dependence to the different environmental conditions (Shepard et al., 2013), namely, obtaining energy (patrolling) and conserving it (traveling), which lead to a favorable net energy gain. The water clarity as well as the bathymetry and the oceanic condition of Guadalupe Island encourages sharks to spend more time patrolling from surface to deep waters, searching for the opportunity to ambush pinniped species inhabiting the island (in most cases the northern elephant seal) and the mesopelagic prey due to the DVM. Since both kinds of prey are sparse and do not congregate in a specific location, sharks may employ a patrolling strategy with greater linearity and fewer turning angles. Meanwhile, the traveling state most widely used by sharks could be employed once they have satisfied their caloric needs, allowing them to focus on moving to areas with physiologically favorable environmental conditions. However, within this category, sharks prefer surface and medium waters over deep waters, presumably due to the less extreme temperatures than those that occur at great depths, which could generate higher metabolic costs (in addition to other potential constraints such as that caused by the low‐oxygen layer, Domeier et al., 2012; Nasby‐Lucas et al., 2009), despite the regional endothermy of white sharks (Afonso et al., 2014; Cartamil et al., 2010; Nasby‐Lucas et al., 2009). For shallow and midrange waters, traveling states presented a homogeneous distribution along the eastern coast of the island. Traveling–Shallow took place mainly throughout the day occurred most intensely in the south corner of the bay, which may correspond to both, a low‐energy lineal displacement in a high current spot, promoting greater oxygenation, or a patrolling hunting behavior since this area is the nearest from the main northern elephant seal beach on the east side of the island. Traveling–Midrange was more concentrated between the two main northern elephant seal colonies, and took place mainly at night and twilight hours. During night time, white sharks could take profit of the DSL rising from 350 to 500 m below to just a range of <200 m; in this sense, Becerril‐García et al. (2020) found squid tentacle fresh scars on the skin of white sharks from Guadalupe Island, with strong evidence that these scars had appeared shortly since last observation; the access to these prey was also found by Papastamatiou et al. (2020) with oceanic whitetip sharks, and Le Croizier et al. (2020) confirmed the importance of mesopelagic prey contribution in Guadalupe's white shark diet. During dawn and sunset, this behavior possibly indicates a patrolling hunting strategy under low‐light conditions, where the silhouette of the prey against the surface is clearer (Domeier et al., 2012; Hoyos‐Padilla et al., 2016; Skomal et al., 2015). Traveling–Deep extended the farthest from the coast, concentrated mainly in the northern third of the eastern side of the island. This behavioral state prevailed during twilight, probably in association with pinniped hunting behavior, and daytime hours, when DSL is deeper, mirroring it as the DVP showed for other pelagic species (Afonso et al., 2014; Jorgensen et al., 2012; Weng et al., 2007).

In contrast to the results of Towner et al. (2016), no effects were observed for sex, individual preferences, or potential habitat in our study. This could be due in part to differences in the trackings carried out: Because of forecast conditions and the study area, Towner et al. (2016) repeatedly tracked tagged sharks for <12 h in sunlight. This allowed them to register individual and sex‐based differences in behavior. Instead, our trackings were longer on average for each animal, even exceeding 24 h in some cases, but less numerous. Since the energy requirements of sharks and environmental variables can vary over time, following the sharks for more continuous hours but fewer days made it impossible to observe these differences. Increasing and homogenizing the number of tracking hours in different lunar and tidal phases will improve the ability of the model to reflect the effects of these variables on the behavioral states of adult and juvenile white sharks of both sexes. White shark tracking also occurred in Gansbaai (a semi‐closed bay) and Dyer and Geyser Rock islands (separated by Shark Alley). This study area had greater heterogeneity than Guadalupe Island; as a result, sharks could adapt their behavior based on the biotic and abiotic factors in each area (energy landscape). Additionally, Towner et al. (2016) were able to record predation events while following some of their specimens. However, the main limitations of this study were the lack of data at night and the missing analysis of behaviors with depth. Although tens of predation events have been observed at Guadalupe Island, especially on elephant seals, these events were detected via observations of sharks feeding on already dead prey, supporting the hypothesis proposed by Hoyos‐Padilla (2009) that sharks attack elephant seals in deep and shadowy waters, ambushing them in canyon areas due to water visibility >30 m (Skomal et al., 2015).

In conclusion, our novel HMM analysis allowed us to decompose the behavior of the animals of this study into other more accurate ones; the results showed 2 main and a total of 6 sub‐behaviors (by the inclusion of a third dimension). As expected, according to the principle of energy conservation, a greater number of behaviors with lower energy costs, the Traveling states, were observed (77.8% overall time), adapted to the existing conditions in the environment; these were more extensive and prolonged in the 3 dimensions, Traveling–Midrange being the most used due to its versatility in the access to the different prey, among other factors. In contrast, ARS (22.2% overall time) was the least used presumably due to being more costly in terms of energy expended. However, due to the attraction effect of the shark cage‐diving operation, ARS–Shallow was the most used in the space‐time in which this activity took place. Being an oceanic island, Guadalupe favors access to mesopelagic prey and the habitat use of twilight zone (200–1000 m); its importance in the white shark's diet is reflected in the results of the present study complementing what has been observed in previous studies under other approaches.

## CONFLICT OF INTERESTS

The authors declare that they have no competing interests.

## AUTHOR CONTRIBUTION

**Marc Aquino‐Baleytó:** Conceptualization (equal); Data curation (equal); Funding acquisition (supporting); Investigation (equal); Methodology (equal); Project administration (supporting); Resources (equal); Writing‐original draft (lead). **Vianey Leos‐Barajas:** Conceptualization (equal); Formal analysis (lead); Investigation (supporting); Methodology (equal); Software (lead); Supervision (equal); Validation (equal); Writing‐original draft (supporting). **Timo Adam:** Conceptualization (supporting); Data curation (supporting); Formal analysis (supporting); Methodology (supporting); Software (supporting); Supervision (supporting); Validation (equal); Visualization (equal); Writing‐review & editing (equal). **Mauricio Hoyos‐Padilla:** Conceptualization (supporting); Funding acquisition (equal); Investigation (lead); Methodology (supporting); Project administration (lead); Resources (equal); Supervision (equal); Validation (equal); Writing‐review & editing (equal). **Omar Santana‐Morales:** Conceptualization (supporting); Data curation (supporting); Funding acquisition (lead); Investigation (equal); Methodology (equal); Project administration (supporting); Resources (equal); Supervision (equal); Writing‐review & editing (supporting). **Felipe Galván‐Magaña:** Investigation (equal); Project administration (equal); Validation (equal); Writing‐review & editing (equal). **Rogelio González‐Armas:** Investigation (supporting); Supervision (equal); Validation (equal); Writing‐review & editing (equal). **Christopher G. Lowe:** Conceptualization (supporting); Investigation (supporting); Supervision (equal); Validation (equal); Visualization (equal); Writing‐review & editing (equal). **James T. Ketchum:** Conceptualization (supporting); Investigation (equal); Project administration (equal); Supervision (equal); Validation (equal); Writing‐review & editing (equal). **Héctor Villalobos:** Conceptualization (supporting); Data curation (equal); Investigation (equal); Methodology (supporting); Project administration (supporting); Resources (supporting); Software (supporting); Supervision (equal); Validation (equal); Visualization (equal); Writing‐review & editing (equal).

## Supporting information

Supplementary Material

## Data Availability

Aquino‐Baleytó, M., Santana‐Morales, O., & Hoyos‐Padilla, E. M. (2021). Shark tracking data. http://doi.org/10.17882/83385.
