# Supplementary material for: Diving deeper into the underlying white shark behaviors at Guadalupe Island, Mexico
Source: Ecol Evol. 2021 Oct 18;11(21):14932–49. doi: 10.1002/ece3.8178 (PMC8571628; doi:10.1002/ece3.8178)
Supplement: Supplementary file 1 — Supplementary Material [file ECE3-11-14932-s001.pdf]

Prior distributions for the horizontal movement HMM

Priors for the step length state-dependent distributions for Sharks 1-4:

$$\mu_{1:4,1} \sim N^{+}(50, 5)$$
$$\mu_{1:4,2} \sim N^{+}(175, 10)$$
$$\mu_{1:4,3} \sim N^{+}(300, 10)$$
$$\boldsymbol{\sigma}_{1:4} \sim T_3^{+}(0, 1)$$

Priors for the step length state-dependent distributions for Sharks 6-10:

$$\mu_{5:10,1} \sim N^{+}(50, 5)$$
$$\mu_{5:10,2} \sim N^{+}(200, 10)$$
$$\boldsymbol{\sigma}_{1:4} \sim T_3^{+}(0, 1)$$

Priors for the unconstrained parameters of the turning angle distribution for Sharks 1-4:

$$x_{1:4,1} \sim N(-0.5, 1)$$
$$x_{1:4,2} \sim N(2, 2)$$
$$x_{1:4,3} \sim N(2, 2)$$
$$y_{1:4,\cdot} \sim N(0, 0.5)$$

Priors for the unconstrained parameters of the turning angle distribution for Sharks 6-10:

$$x_{5:10,1} \sim N(-0.5, 1)$$
$$x_{5:10,2} \sim N(2, 2)$$
$$y_{5:10,\cdot} \sim N(0, 0.5)$$

Priors for the parameters of the transition probability matrix:

$$\beta_0^{(i,j)} \sim N(-3, 0.5)$$
$$\beta_{\boldsymbol{c}}^{(i,j)} \sim N(0, 0.5)$$

Priors for the initial state distribution:

$$\boldsymbol{\delta} \sim Dirichlet(\mathbf{1})$$

Prior distributions for the vertical movement HMM

Priors for the depth state-dependent distributions:

$$\mu_1 \sim N^+(2, 1)$$
$$\mu_2 \sim N^+(20, 1)$$
$$\mu_3 \sim N^+(40, 3)$$
$$\mu_4 \sim N^+(70, 3)$$
$$\mu_5 \sim N^+(120, 10)$$
$$\mu_6 \sim N^+(200, 10)$$
$$\boldsymbol{\sigma} \sim N(0, 1)$$

Priors for the parameters of the transition probability matrix:

$$\beta_0^{(i,j)} \sim N(-3, 0.5)$$
$$\beta_{\boldsymbol{c}}^{(i,j)} \sim N(0, 0.5)$$

Priors for the initial state distribution:

$$\boldsymbol{\delta} \sim Dirichlet(\mathbf{1})$$
